# Association between the TLR2 Arg753Gln polymorphism and the risk of sepsis: a meta-analysis

**DOI:** 10.1186/s13054-015-1130-3

**Published:** 2015-11-30

**Authors:** Jun-wei Gao, An-qiang Zhang, Xiao Wang, Zhong-yun Li, Jian-hua Yang, Ling Zeng, Wei Gu, Jian-xin Jiang

**Affiliations:** State Key Laboratory of Trauma, Burns and Combined Injury, Institute of Surgery Research, Daping Hospital, Third Military Medical University, Chongqing, China; The First Affiliated Hospital of Wenzhou University, Wenzhou, Zhejiang Province China

## Abstract

**Introduction:**

Recently, researchers in a number of studies have explored the association between the Toll-like receptor 2 (TLR2) Arg753Gln polymorphism and sepsis risk. However, the results were conflicting. In this meta-analysis, we aimed to confirm the effect of the TLR2 Arg753Gln polymorphism on sepsis risk.

**Methods:**

Relevant records up to 1 June 2015 were retrieved from the PubMed, Embase, and Web of Knowledge databases. The odds ratios with their corresponding 95 % confidence intervals were used to assess the association between the TLR2 Arg753Gln polymorphism and sepsis risk. The selection of a fixed or random effects model was made according to a heterogeneity test in total and subgroup analyses. Sensitivity analysis and publication bias test were performed to ensure the reliability of our results.

**Results:**

A total of 12 studies with aggregate totals of 898 cases and 1517 controls met our inclusion criteria for meta-analysis. There were significant associations between the TLR2 Arg753Gln polymorphism and sepsis risk in overall analyses under two genetic models (the allele comparison and the dominant model). In addition, subgroup analyses based on age group, ethnicity, sepsis type, and source of control also showed a significant effect of the TLR2 Arg753Gln polymorphism on sepsis risk.

**Conclusions:**

Our present meta-analysis supports a direct effect of the TLR2 Arg753Gln polymorphism on sepsis risk, especially in Europeans. The TLR2 Arg753Gln polymorphism might be used as a relevant risk estimate for the development of sepsis. Studies with larger sample sizes and homogeneous groups of patients with sepsis are required for further analysis.

**Electronic supplementary material:**

The online version of this article (doi:10.1186/s13054-015-1130-3) contains supplementary material, which is available to authorized users.

## Introduction

Sepsis is a complex clinical syndrome that results from a systemic inflammatory response to bacteria and/or bacterial products [1]. It remains a leading cause of death in the non-cardiac intensive care unit (ICU) despite improvements in antibiotic therapy and supportive care [2, 3]. Therefore, early identification of patients with a high risk of sepsis after ICU admission is urgently needed to help determine therapeutic interventions. The host innate immune system plays a key role in the development of sepsis [4]. Recently, a number of studies have been conducted to assess the effect of factors in innate immune system on the susceptibility and outcome of sepsis [5–7]. Among these factors, Toll-like receptors (TLRs) have been studied extensively.

TLRs, which are a group of pattern recognition receptors, are composed of ten transmembrane proteins in humans [8] and are expressed mainly on immune cells, such as macrophages and dendritic, B, T, and some non-immune cells [9]. Their important roles have been confirmed in regulating inflammatory reactions and activating adaptive immune response to eliminate infectious pathogens [10]. TLR2, a key member of TLR family, could recognize a variety of bacterial lipoproteins. Several studies have considered TLR2 as the initial barrier against infection [11, 12]. The mechanism of TLR2-recognizing lipoproteins has been elucidated. After TLR2 recognizes lipoproteins, it activates MyD88 adaptor–like protein and triggers a signaling pathway, which induces further immune response [13, 14]. In addition, the TLR2 signaling pathway is essential to systemic inflammation, which has been demonstrated in mice with *Staphylococcus aureus* sepsis [15]. This evidence suggests that *TLR2* may be an appealing candidate gene for determining sepsis risk.

The *TLR2* gene, mapped to chromosome 4q32, consists of three exons. Population-based case–control studies have shown that the polymorphisms of TLR2 could influence poor outcomes in a number of diseases, such as cancer, tuberculosis, and infective endocarditis [16–19]. Among these polymorphisms, the TLR2 Arg753Gln polymorphism (R753Q, rs5743708), a missense single-nucleotide polymorphism, has been the most widely discussed. A previous study suggested that TLR2 Arg753Gln could lead to diminished activation of intracellular signaling pathways [20]. Recently, a large number of studies have been conducted to explore the association between the TLR2 Arg753Gln polymorphism and sepsis risk. However, the results were inconsistent. Thus, we performed a meta-analysis to further investigate the effect of the TLR2 Arg753Gln polymorphism on sepsis risk.

## Material and methods

### Identification and eligibility of relevant studies

We searched all published articles in the PubMed, Embase, and Web of Knowledge databases up to 1 June 2015. The keywords used were as follows: “Toll-like receptor 2” or “TLR2”; “sepsis” “septic shock,” or “severe sepsis”; and “polymorphism,” “variation,” “mutation,” or “genotype.” Relevant studies were retrieved, and their references were checked to assess other relevant publications. Authors were contacted to obtain related data not revealed in the original articles.

The inclusion criteria were as follows: (1) the study had to include an evaluation of the association between the TLR2 Arg753Gln polymorphism and sepsis risk; (2) the study design had to be a case–control or cohort study; and (3) the number of the study population genotypes had to be stated in the article or obtained by contacting the authors. The exclusion criteria were as follows: (1) study with insufficient number of population genotype; (2) review, comment, or abstract; and (3) no mutation in the study population. When an article reported results on subjects of different ethnicities, we treated each ethnicity as a separate study.

### Data extraction

Two investigators independently screened titles, abstracts, and full text of the articles to reduce errors and ensure the reliability of the results. A standardized extraction form was used for information collection. Disagreement was resolved by discussion. Information collected from the selected studies include the first author’s name, publication year, country in which the study was done, subjects’ ethnicities, subjects’ age groups, sepsis types, source of control subjects, and genotype numbers in cases and control subjects for each TLR2 Arg753Gln genotype. Hardy-Weinberg equilibrium (HWE) was tested based on the collected data.

### Statistical analysis

We performed our meta-analysis under the allele comparison model (A vs. B) and the dominant model (AB/AA vs. BB) for the unknown inherited model of sepsis. Because of the lack of a mutant homozygote for the TLR2 Arg753Gln polymorphism, we could not conduct the related analysis under other inherited models. Odds ratios (ORs) with their 95 % confidence intervals (CIs) were used to assess the association between the TLR2 Arg753Gln polymorphism and sepsis risk. The Z-test was selected to evaluate the statistical significance of pooled ORs. In addition to total comparisons, stratified analyses based on age group, ethnicity, sepsis type, and source of control subjects were also carried out. Heterogeneity across studies was evaluated with the *I*^2^ value and a χ^2^-based Q test. *P* > 0.10 for the Q test and *I*^2^ values less than 50 % revealed no obvious heterogeneity across studies, allowing us to use a fixed effects model (the Mantel-Haenszel method); otherwise, a random effects model (the DerSimonian and Laird method) was selected. Galbraith plots were used to investigate the source of between-study heterogeneity. Sensitivity analysis was performed by sequentially removing individual studies to assess the reliability of our results. Publication bias was examined by Begg’s funnel plot qualitatively (the more symmetrical, the lower the risk of publication bias) and Egger’s test quantitatively. All statistical analyses were performed using RevMan 5.2 software (Nordic Cochrane Center, Copenhagen, Denmark) and STATA 12.0 software (StataCorp, College Station, TX, USA).

## Results

### Characteristics of eligible studies

**Fig. 1:**
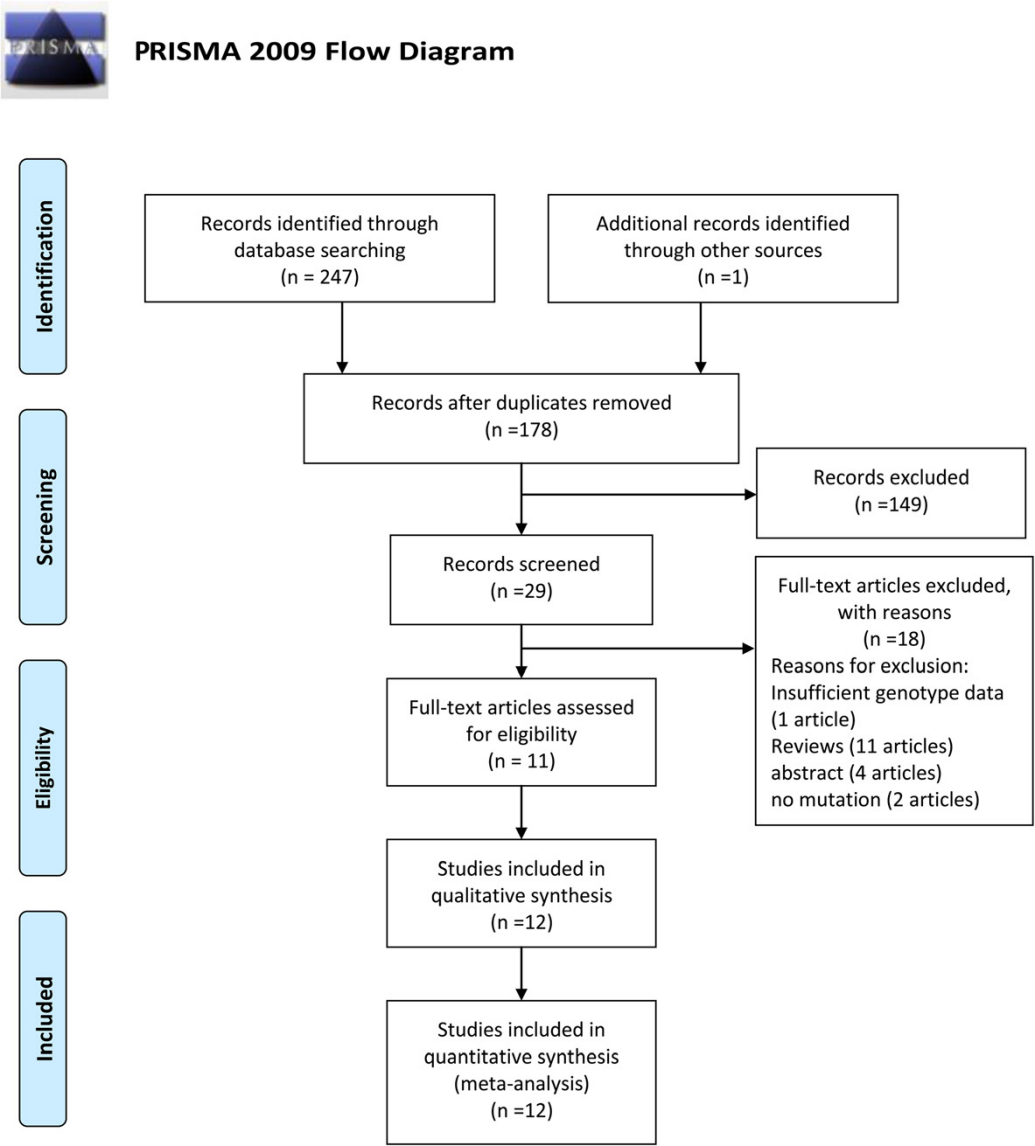
Flow diagram of study identification. Because the study conducted by McDaniel et al. [29] reported results on populations of different ethnicities, we treated the populations as two separate studies (study 1 and study 2). A total of 12 studies (11 articles) were ultimately included in our meta-analysis.

A total of 247 records were identified by using different combinations of search terms in PubMed, Embase, and Web of Knowledge, and 1 record was identified by checking reference lists. After excluding 70 duplications, 149 records were removed for their unmatched titles or abstracts. Full-text reading helped us to remove 18 records (1 record with insufficient genotype data, 11 reviews, 2 records without mutation genotype, and 4 meeting abstracts). The author of record with insufficient genotype data was contacted. However, no answer was received. Ultimately, 12 studies (11 records) [21–31] were included in our meta-analysis according to the inclusion and exclusion criteria we had set. The study selection process is shown in Fig. 1.

Of the 12 included studies, 9 were conducted with Europeans, 1 in an Asian Han population, 1 in an African population, and 1 in a mixed ethnicities population. Seven studies were about sepsis, one was on severe sepsis, and one was about septic shock. In addition, the study populations in our included studies consisted of adults (nine studies) and children (three studies). Five studies used critically ill patients and another five used healthy populations as control subjects. All of the studies were consistent with HWE, except for the study conducted by Lee et al. [25]. The characteristics of selected studies are presented in Table 1.

**Table 1:** Characteristics of the studies included in the meta-analysis

Study	Country	Ethnicity	Age group	Sepsis type	Source of control subjects	Sample size		Case polymorphisms			Control polymorphisms			HWE
						Cases	Controls	GG	GA	AA	GG	GA	AA	
Schnetzke et al., 2015 [21]	Germany	European	Adult	Sepsis	Patients with acute myeloid leukemia	74	81	66	7	1	79	2	0	Yes
Tellería-Orriols et al., 2014 [22]	Spain	European	Pediatric	Sepsis	Healthy	153	66	61	72	20	49	15	2	Yes
Nachtigall et al., 2014 [23]	Germany	European	Adult	Mix	Critically ill patients	98	47	88	10	0	47	0	0	Yes
Bronkhorst, et al., 2013 [24]	The Netherlands	European	Adult	Sepsis	Critically ill patients	79	140	74	5	0	130	10	0	Yes
Lee et al., 2011 [25]	United States	European	Adult	Mix	Patients after liver transplant	187	405	169	15	3	366	27	12	No
Ahmad-Nejad et al., 2011 [26]	Germany	European	Adult	Mix	Critically ill patients	38	112	34	4	0	107	5	0	Yes
Shan et al., 2010 [27]	China	Han Chinese	Pediatric	Severe sepsis	Healthy	38	57	36	2	0	57	0	0	Yes
Davis et al., 2010 [31]	United States	European	Adult	Sepsis	Healthy	24	53	19	5	0	48	5	0	Yes
Yuan et al., 2008 [28]	Australia	Mix	Pediatric	Sepsis	Healthy	85	409	82	3	0	382	27	0	Yes
McDaniel et al., 2007 [29] (study 2)	United States	European	Adult	Sepsis	Critically ill patients	15	21	12	3	0	17	4	0	Yes
McDaniel et al., 2007 [29] (study 1)	United States	African	Adult	Sepsis	Critically ill patients	16	16	6	10	0	12	4	0	Yes
Lorenz et al., 2000 [30]	France	European	Adult	Septic shock	Healthy	91	110	89	2	0	107	3	0	Yes

*HWE* Hardy-Weinberg equilibrium

### Quantitative data synthesis

**Fig. 2:**
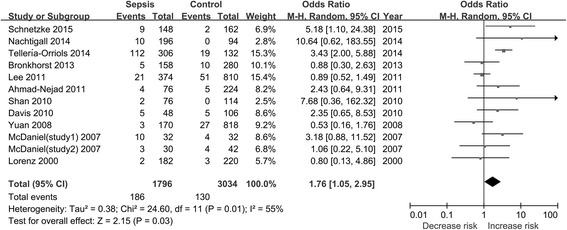
Forest plot of sepsis risk associated with the Toll-like receptor 2 Arg753Gln polymorphism in the allele comparison model. “Total” in this figure means the number of alleles in the corresponding group. *CI* confidence interval, *M-H* Mantel-Haenszel

**Fig. 3:**
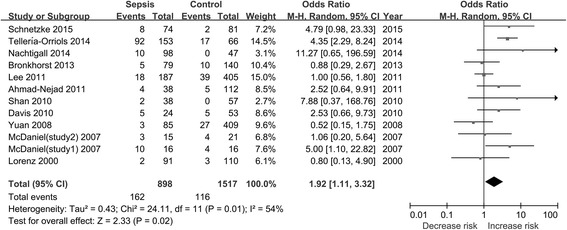
Forest plot of sepsis risk associated with the Toll-like receptor 2 Arg753Gln polymorphism in the dominant model. “Total” in this figure means the number of people in the corresponding group. *CI* confidence interval, *M-H* Mantel-Haenszel

A total of 12 studies (11 records) with 898 cases and 1517 controls were examined to determine the association between the TLR2 Arg753Gln polymorphism and sepsis risk [21–31]. The combined results of the overall comparison indicated that there were significant associations between the TLR2 Arg753Gln polymorphism and sepsis risk under the allele comparison model and the dominant model, respectively (for A vs. G, OR 1.76, 95 % CI 1.05–2.95, *P* = 0.03; for AA/GA vs. GG, OR 1.92, 95 % CI 1.11–3.32, *P* = 0.02) (Table 2, Figs. 2 and 3). After removing the study that was not consistent with HWE [25], the results remained similar (data not shown). In addition, subgroup analyses showed significant associations between the TLR2 Arg753Gln polymorphism and sepsis risk in the adult group. Also, the TLR2 Arg753Gln polymorphism increased sepsis risk in the European and critically ill patient subgroups, respectively. The results of our meta-analysis are presented in Table 2.

**Table 2:** Summary of meta-analysis results

0		Tests of association				Tests of heterogeneity		
Groups	Studies, n	OR (95 % CI)	*P* value	Model	Z	χ^2^	*P* value	*I* ^2^ (%)
All studies								
A vs. G	12	1.76 (1.05–2.95)	0.03	RE	2.15	24.60	0.01	55
AA/GA vs. GG	12	1.92 (1.11–3.32)	0.02	RE	2.33	24.11	0.01	54
Subgroup analyses								
Adult								
A vs. G	9	1.42 (1.00–1.99)	0.05	FE	1.99	11.68	0.17	32
AA/GA vs. GG	9	1.57 (1.09–2.28)	0.02	FE	2.39	10.97	0.20	27
Pediatric								
A vs. G	3	1.94 (0.42–8.89)	0.39	RE	0.86	8.29	0.02	76
AA/GA vs. GG	3	2.18 (0.39–12.08)	0.37	RE	0.89	9.75	0.008	79
European								
A vs. G	9	1.81 (1.02–3.23)	0.04	RE	2.02	19.18	0.01	58
AA/GA vs. GG	9	1.95 (1.08–3.51)	0.03	RE	2.22	17.43	0.03	54
Sepsis								
A vs. G	7	1.86 (0.99–3.52)	0.05	RE	1.93	13.06	0.04	54
AA/GA vs. GG	7	2.01 (0.97–4.19)	0.06	RE	1.87	15.15	0.02	60
Critically ill patients								
A vs. G	5	1.95 (1.08–3.51)	0.03	FE	2.22	4.63	0.33	14
AA/GA vs. GG	5	2.09 (1.13–3.86)	0.02	FE	2.34	5.64	0.23	29
Healthy								
A vs. G	5	1.76 (0.71–4.35)	0.22	RE	1.22	9.78	0.04	59
AA/GA vs. GG	5	1.88 (0.67–5.25)	0.23	RE	1.20	11.42	0.02	65
HWE								
A vs. G	11	2.00 (1.20–3.34)	0.008	RE	2.66	16.12	0.10	38
AA/GA vs. GG	11	2.15 (1.20–3.84)	0.01	RE	2.59	18.23	0.05	45

*RE* random effects model, *FE* fixed effects model, *HWE* Hardy-Weinberg equilibrium

### Heterogeneity analysis

Statistically significant between-study heterogeneity was found in overall comparisons in the allele comparison model (*P* = 0.01) and the dominant model (*P* = 0.01), respectively. Subgroup analyses were performed to ensure the homogeneity of study populations. However, the between-study heterogeneity did not decrease substantially, except in adult and critically ill patient subgroup analyses. A Galbraith plot was selected to explore the source of heterogeneity for total analyses. The studies conducted by Lee et al. [25] and Tellería-Orriols et al. [22] were the outliers in the Galbraith plot using the allele comparison model (Additional file 1). After removing those studies, the between-study heterogeneity decreased substantially and there was no obvious heterogeneity among the remaining studies (*P* = 0.21, *I*^2^ = 25 %). The association between the TLR2 Arg753Gln polymorphism and sepsis risk changed little (for A vs. G, OR 1.74, 95 % CI 1.15–2.63, *P* = 0.009) (Additional file 2). In addition, the study performed by Tellería-Orriols et al. [22] was outside the bounds in Galbraith plot in the dominant model (Additional file 3). After excluding that study, the between-study heterogeneity decreased notably and no significant between-study heterogeneity was found among the remaining studies (*P* = 0.14, *I*^2^ = 32 %). There was a significant association between the TLR2 Arg753Gln polymorphism and sepsis risk (for AA/GA vs. GG, OR 1.44, 95 % CI 1.02–2.03, *P* = 0.04) (Additional file 4). The data derived from the heterogeneity analysis are shown in Table 2.

### Sensitivity analysis

Sensitivity analysis was performed to evaluate the influence of each individual study on pooled ORs by removing each study sequentially. No obvious changes were found in the results, which confirmed the reliability of our results in two genetic models.

### Publication bias

**Fig. 4:**
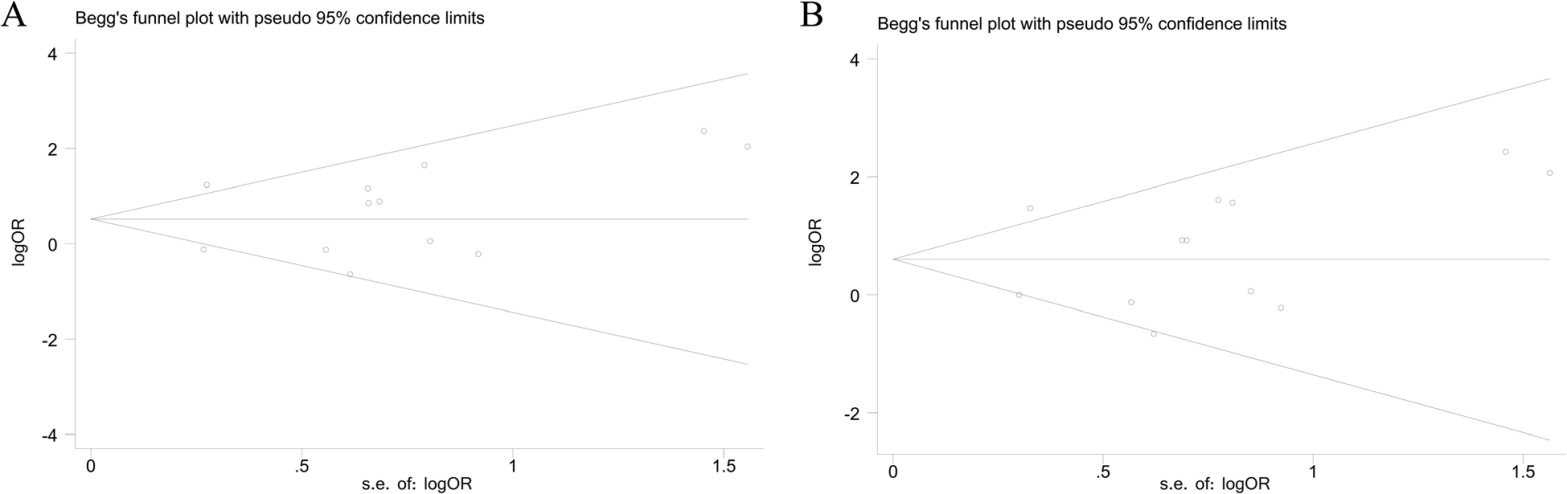
Funnel plots of the Toll-like receptor 2 Arg753Gln polymorphism and the risk of sepsis to assess publication bias in different models. **a** The allele comparison model. **b** The dominant model. OR odds ratio

Publication bias within each study might not represent all studies. Therefore, Egger’s test and Begg’s funnel plot were used to evaluate publication bias quantitatively and qualitatively, respectively. Although slightly asymmetrical funnel plots were found in our results (Fig. 4), Egger’s test did not exhibit obvious publication bias in the allele comparison model (*P* = 0.599) and the dominant model quantitatively (*P* = 0.590).

### Discussion

Sepsis is a complex clinical syndrome that results from a systemic inflammatory response to bacteria and/or bacterial products. Previous studies have shown that the innate immune system is an essential part of sepsis development and progression [4, 32, 33]. TLRs and their associated downstream regulators of immune cell functions play a crucial role in the innate system as the first line of defense against pathogens. Among all mammalian TLRs, TLR2 is capable of detecting the widest repertoire of pathogen-associated molecular patterns. Differences in polymorphism-associated TLR2 may influence the recognition of pathogens, which could cause the different degrees of the host response [34]. Further, the TLR2 Arg753Gln polymorphism was associated with an increased risk of developing tuberculosis [35]. Therefore, we hypothesized that the TLR2 Arg753Gln polymorphism could affect the development of sepsis.

To the best of our knowledge, this is the first meta-analysis in which the relationship between TLR2 polymorphism and sepsis risk has been explored. We found significant associations between the TLR2 Arg753Gln polymorphism and sepsis risk in two genetic models (the allele comparison model and the dominant model). In addition, subgroup analyses based on age group, ethnicity, sepsis type, and source of control subjects were performed. We noted that the TLR2 Arg753Gln polymorphism was also significantly associated with sepsis risk in adult, European, sepsis, and critically ill patient subgroups. These results further proved the important role of the TLR2 Arg753Gln polymorphism in the development of sepsis. Since there were fewer than three studies performed in other subgroups, analyses of other subgroups were not conducted; more studies are required to analyze these conditions.

Our meta-analysis included a study with patients taking immune-suppressive medications after liver transplant, and the results also were not inconsistent with HWE. After we removed that study from the overall analysis, the results changed little. These patients may not have influenced our results, and more patients taking immune-suppressive medications are needed to explore this association. Some researchers stated that they believe that pediatric patients may be more sensitive than adult patients to sepsis risk factors. However, there was no significant association between the TLR2 Arg753Gln polymorphism and susceptibility to sepsis in our pediatric subgroup analysis. This finding may be due to a relatively small sample size in our meta-analysis. In addition, we found that only one study was performed in an Asian population in our meta-analysis. More studies in Asian populations are needed to estimate the effect of the TLR2 Arg753Gln polymorphism on sepsis risk.

To better ensure the reliability of our results, we also explored the source of between-study heterogeneity in our meta-analysis. Obvious between-study heterogeneity was found in both total and subgroup analyses. Galbraith plots indicated that the studies conducted by Lee et al. [25] and Tellería-Orriols et al. [22] may have been the main source of between-study heterogeneity. The potential bias of these studies might result from the populations studied, the studies’ research methods, or some unknown reasons. After removing these two studies from the overall analysis, the between-study heterogeneity decreased substantially and the association between the TLR2 Arg753Gln polymorphism and sepsis risk was still significant. Moreover, we conducted sensitivity analyses in our meta-analysis. Publication bias could have suppressed false-negative results or could have magnified false-positive results. In our results, the shape of Begg’s funnel plots was slightly asymmetrical, but Egger’s test revealed no obvious publication bias for sepsis risk. All these results made our conclusions stronger.

How might the TLR2 Arg753Gln polymorphism affect susceptibility to sepsis? This non-synonymous arginine-to-glutamine substitution occurs in the intracellular Toll/interleukin 1 receptor domain of the *TLR2* gene. A gene function study indicated that the TLR2 Arg753Gln polymorphism could impair tyrosine phosphorylation, dimerization with TLR6, and MyD88 recruitment with an effect on nuclear factor κB activation [20, 30]. These actions result in defective intracellular signaling and impaired cytokine secretion in response to peptidoglycan, lipopeptides, and other known ligands, which may contribute to the development of sepsis. In addition, animal models have revealed that the defective TLR2 signaling is a causative factor for increased susceptibility to bacterial disease. All of these findings are in agreement with our results, indicating that the TLR2 Arg753Gln polymorphism may affect the risk of sepsis.

Some limitations of our meta-analysis should be pointed out. First, the number of included studies and the sample sizes were moderate, and the heterogeneity was still existed in some subgroups in our meta-analysis, which may have contributed to the modest results. Some studies without sufficient information were excluded, and some subgroup analyses were not conducted in the small number of studies. Second, sepsis is a complex syndrome concerning different pathogens, different ethnicities, different age groups, and different underlying diseases. We could take only some of them into consideration because of their limited information. Third, the study population in our meta-analysis was focused on people of European ethnicity. Our conclusions may be not generalizable to Asian populations. Fourth, many genes are associated with sepsis. However, we could not address gene–gene interactions in this meta-analysis, owing to the lack of related information. Fifth, selection bias may exist because negative studies are difficult to publish. Sixth, the quality of our included studies was not assessed for their limited information. Seventh, we could discuss only the association between the TLR2 Arg753Gln polymorphism and sepsis risk in two genetic models for the lack of a mutant homozygote.

## Conclusions

In our meta-analysis, we pooled all available data related to potential links between the TLR2 Arg753Gln polymorphism and sepsis risk. The evidence suggested that the TLR2 Arg753Gln polymorphism could increase sepsis risk, especially in European populations, which may help us identify high-risk patients. Future large, well-designed epidemiological studies are required to validate this conclusion.

## Key messages

- Previous studies showed conflicting results regarding the effect of the TLR2 Arg753Gln polymorphism on sepsis risk.
- To the best of our knowledge, this is the first meta-analysis in which the relationship between TLR2 polymorphism and sepsis risk was explored.
- In our meta-analysis, we found that the TLR2 Arg753Gln polymorphism could increase sepsis risk, especially in European populations, based on previous studies.

## Additional files

Additional file 1:
**Galbraith plot of the TLR2 Arg753Gln polymorphism and the risk of sepsis under the allele comparison model.** (TIF 441 kb) Additional file 2:
**Forest plot of sepsis risk associated with TLR2 Arg753Gln under the allelic comparison model after deleting the studies conducted by Lee et al. [** 25 **] and Tellería-Orriols et al. [** 22 **].** “Total” in this figure means the number of allele in the corresponding group. (TIF 867 kb) Additional file 3:
**Galbraith plot of the TLR2 Arg753Gln polymorphism and the risk of sepsis under the dominant model.** (TIF 441 kb) Additional file 4:
**Forest plot of sepsis risk associated with TLR2 Arg753Gln under the dominant model after deleting the study conducted by Tellería-Orriols et al. [** 22 **].** “Total” in this figure means the number of people in the corresponding group.References. (TIF 897 kb)

## Abbreviations

CI: confidence interval
HWE: Hardy-Weinberg equilibrium
ICU: intensive care unit
OR: odds ratio
TLR2: Toll-like receptor 2
