# Supplementary figures and images for: Association between the TLR2 Arg753Gln polymorphism and the risk of sepsis: a meta-analysis
Source: Crit Care. 2015 Nov 30;19:416. doi: 10.1186/s13054-015-1130-3 (PMC4663740; doi:10.1186/s13054-015-1130-3)

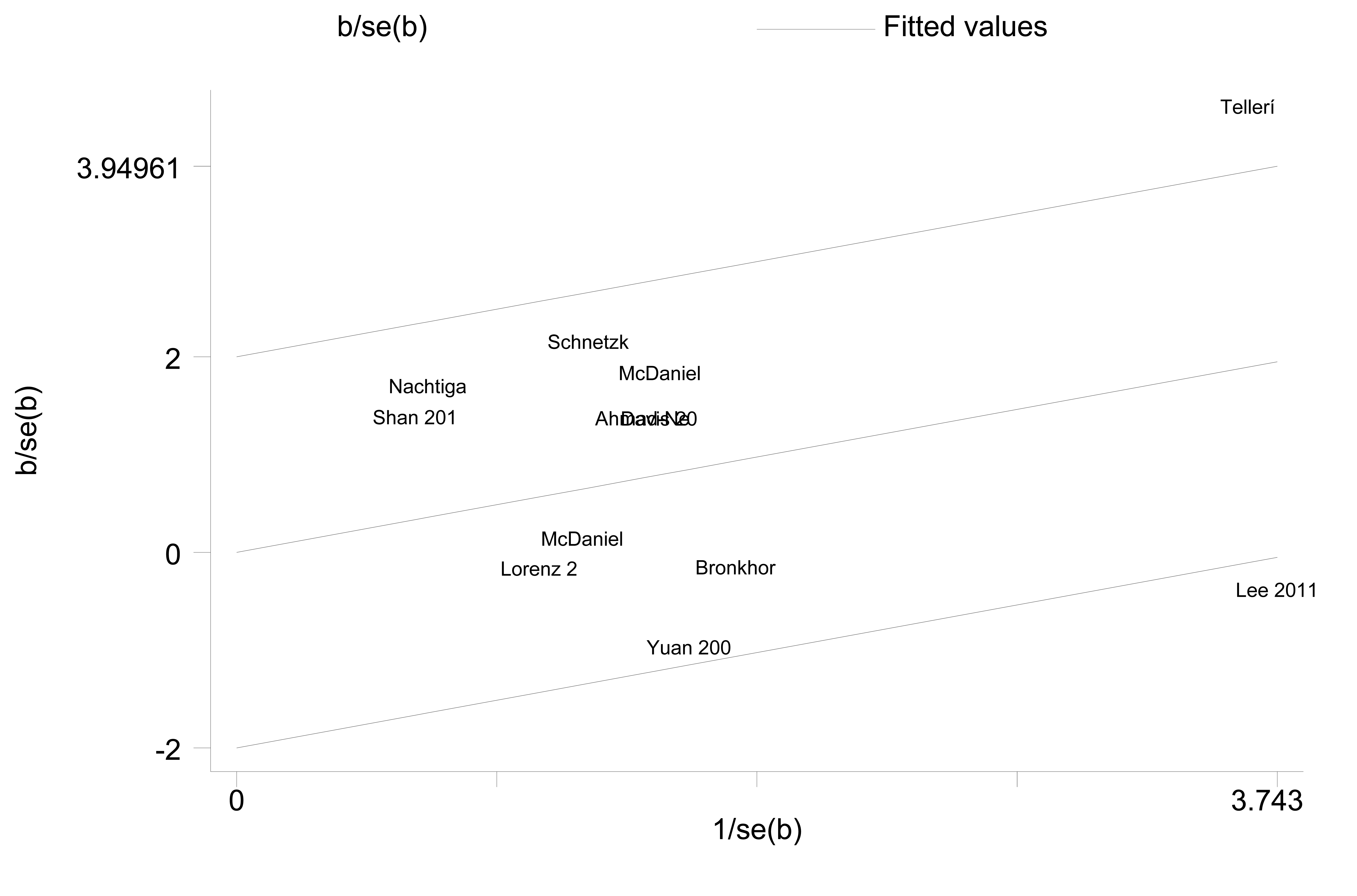

Supplement: Additional file 1: — Galbraith plot of the TLR2 Arg753Gln polymorphism and the risk of sepsis under the allele comparison model. (TIF 441 kb) [file 13054_2015_1130_MOESM1_ESM.tif]

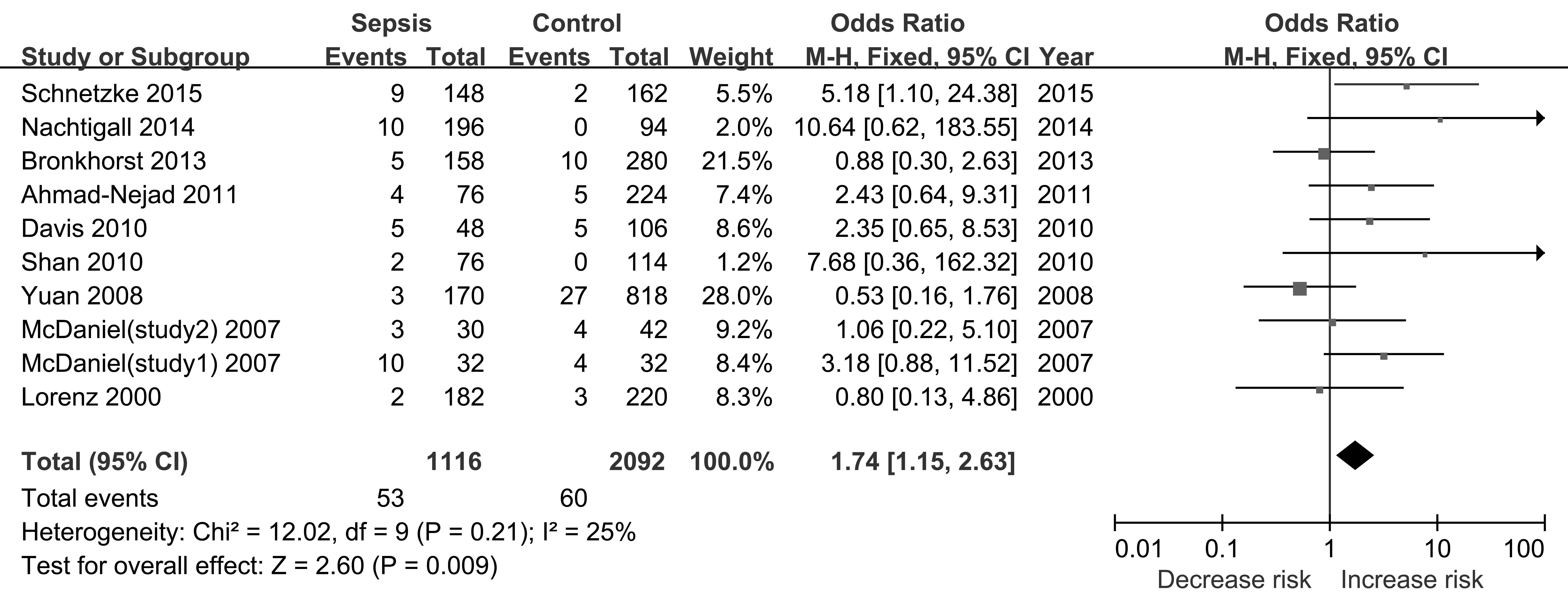

Supplement: Additional file 2: — Forest plot of sepsis risk associated with TLR2 Arg753Gln under the allelic comparison model after deleting the studies conducted by Lee et al. [ 25 ] and Tellería-Orriols et al. [ 22 ]. “Total” in this figure means the number of allele in the corresponding group. (TIF 867 kb) [file 13054_2015_1130_MOESM2_ESM.tif]

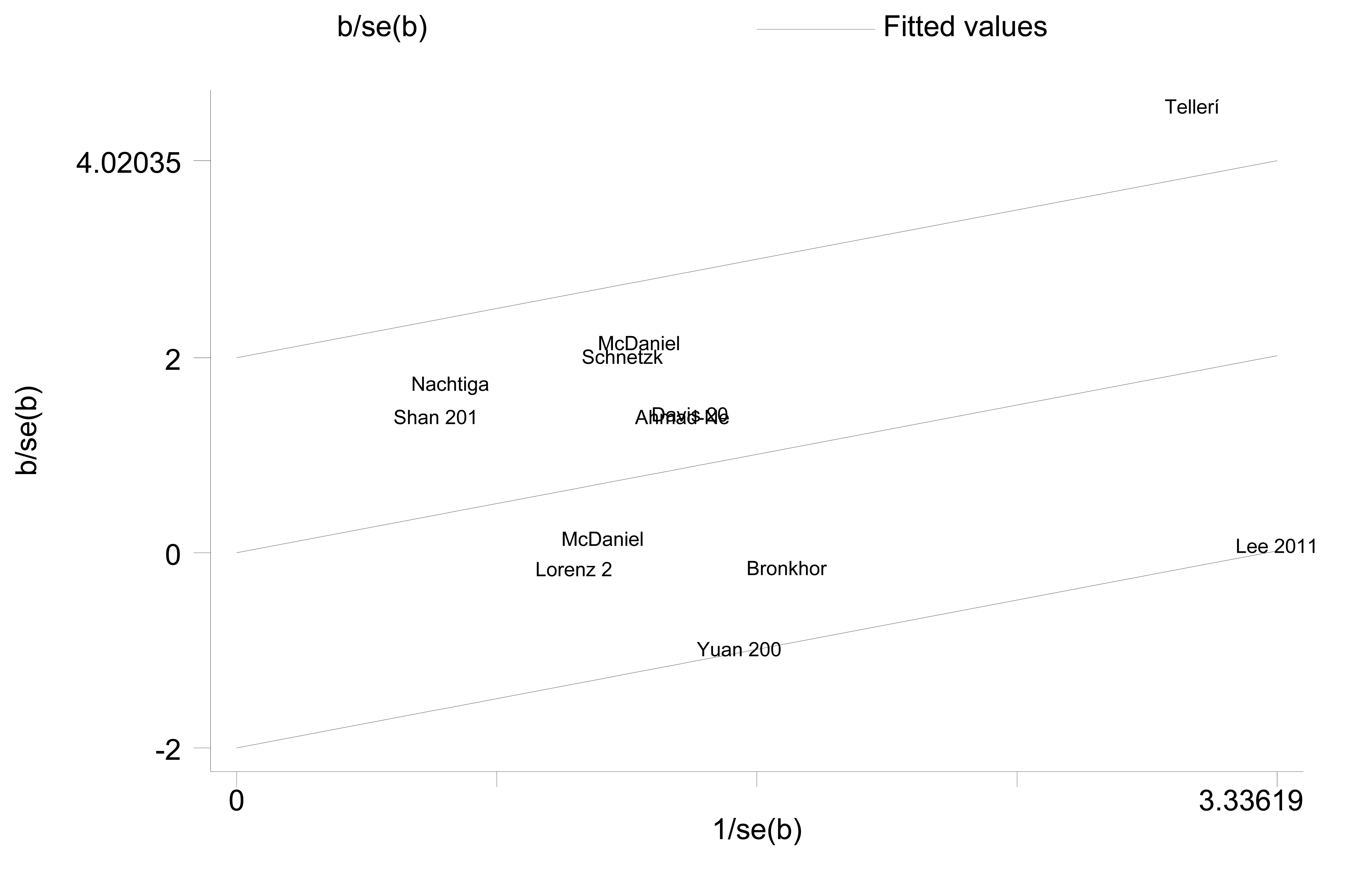

Supplement: Additional file 3: — Galbraith plot of the TLR2 Arg753Gln polymorphism and the risk of sepsis under the dominant model. (TIF 441 kb) [file 13054_2015_1130_MOESM3_ESM.tif]

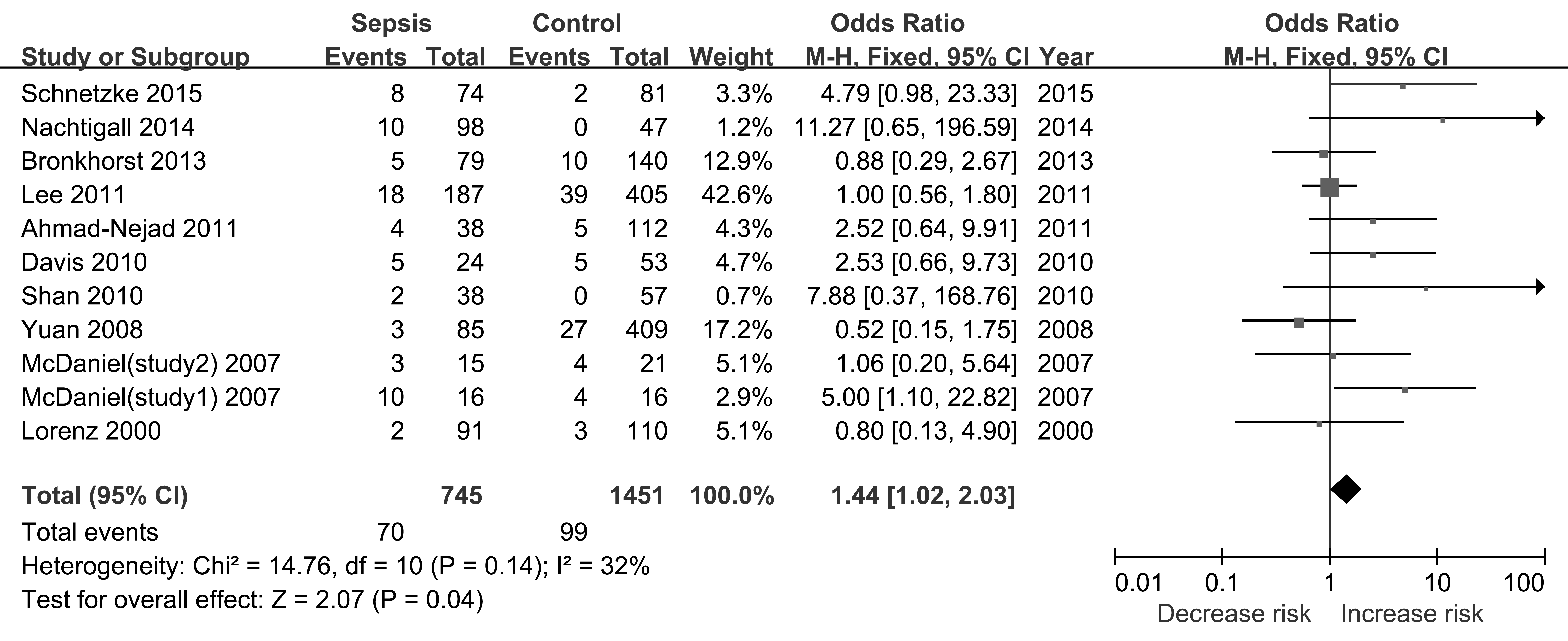

Supplement: Additional file 4: — Forest plot of sepsis risk associated with TLR2 Arg753Gln under the dominant model after deleting the study conducted by Tellería-Orriols et al. [ 22 ]. “Total” in this figure means the number of people in the corresponding group.References. (TIF 897 kb) [file 13054_2015_1130_MOESM4_ESM.tif]
